# Selenium-rich food potentially useful to control mercury levels among Afro-Colombians: Towards an intercultural intervention

**DOI:** 10.7705/biomedica.6981

**Published:** 2023-12-01

**Authors:** Sonia M. Díaz, Ruth Marién Palma, Edna M. Gamboa, Álvaro J. Idrovo

**Affiliations:** 1 Departamento de Salud Pública, Escuela de Medicina, Universidad Industrial de Santander, Bucaramanga, Colombia Universidad Industrial de Santander Universidad Industrial de Santander Bucaramanga Colombia; 2 Grupo de Salud Ambiental y Laboral, Instituto Nacional de Salud, Bogotá, D.C., Colombia Instituto Nacional de Salud Bogotá D.C. Colombia; 3 Escuela de Nutrición y Dietética, Universidad Industrial de Santander, Bucaramanga, Colombia Universidad Industrial de Santander Universidad Industrial de Santander Bucaramanga Colombia

**Keywords:** mercury, selenium, mining, diet, ethnicity, mercurio, selenio, minería, dieta, etnicidad

## Abstract

**Introduction.:**

Diet-based interventions may be a culturally acceptable option to decrease mercury levels and thus prevent the adverse effects of this metal on population health. Selenium is an element present in Colombian geology that can act as a chelator, decreasing mercury concentrations in the human body.

**Objective.:**

To identify potentially useful selenium-rich foods to control the effects of mercury exposure among Afro-Colombians.

**Materials and methods.:**

A cross-sectional study was carried out with 320 individuals from five municipalities of Chocó. They were asked about the frequency of consumption of selenium-rich foods, and their association with mercury concentrations in hair was estimated with multiple robust regression.

**Results.:**

Guava, whole wheat flour, strawberries, cow liver, spinach and yeast extract were the foods with higher consumption. Walnuts, whole wheat flour, and yeast extract were identified in multiple robust regression as foods to consider in future interventions.

**Conclusion.:**

It is proposed that the banana juice, the pineapple colada, the borojó (Borojoa patinoi) sorbet, the cucas, and the enyucado are basic elements for a culturally acceptable intervention.

## Resumen

**Introducción.:**

Las intervenciones basadas en la dieta pueden ser una opción culturalmente aceptable para disminuir los niveles de mercurio y prevenir los efectos adversos de este metal en la salud de la población. El selenio es un elemento presente en la geología colombiana que puede actuar como quelante, disminuyendo las concentraciones de mercurio en el cuerpo humano.

**Objetivo.:**

Identificar los alimentos ricos en selenio potencialmente útiles para controlar los efectos de la exposición a mercurio entre afrocolombianos.

**Materiales y métodos.:**

Se realizó un estudio transversal con 320 individuos de cinco municipios del departamento del Chocó. Se les preguntó sobre la frecuencia de consumo de alimentos ricos en selenio y su asociación con las concentraciones de mercurio en cabello fue estimada con regresión múltiple robusta.

**Resultados.:**

La guayaba, la harina de trigo integral, las fresas, el hígado de vaca, la espinaca y el extracto de levadura fueron los alimentos de mayor consumo. Las nueces, la harina de trigo integral y el extracto de levadura fueron identificados en regresión múltiple robusta como alimentos por considerar en futuras intervenciones.

**Conclusiones.:**

Se propone que el jugo de plátano, la piña colada, el sorbete de borojó (Borojoa patinoi), las cucas, el enyucado y la sábida de plátano primitivo maduro son elementos básicos para una intervención culturalmente aceptable.

Exposure to mercury in gold mining contexts is frequent and risky to human health, especially in artisanal and small-scale mines in African, Asian, and Latin American countries ^1^. In these contexts, environmental and occupational exposures are high and frequent, and related with sociocultural process of vulnerable populations. Higher levels of the trophic chain are related with the highest concentrations of mercury as consequence of bioaccumulation and biomagnification processes ^2^. For this reason, humans and other carnivorous species are more exposed to mercury. However, special attention has been given to neonates and children due to the their high biological susceptibility related with the presence of methyl-mercury in foods (fish and other marine animals) from rivers, sea, or other water sources ^3^^,^^4^.

Although there are several experiences with interventions to reduce the effects of mercury exposure, the results have not been consistent ^5^. This suggests that interventions should be tailored to each population. In Latin America, some dietary interventions have been proposed to reduce the potential effects of environmental exposure to mercury. Most have focused on reducing the consumption of fish, or carnivorous fish ^6^, or to increase the consumption of selenium-rich foods ^7^. However, there is another group of interventions that suggest reducing breastfeeding ^8^, without considering the conditions of social vulnerability associated with poverty in populations exposed to mercury. In general, these interventions are focused on reducing exposure to organic mercury of dietary origin, without considering the occupational or para-occupational exposure that can occur in these places ^9^.

Unfortunately, proposals of this type can lead to malnutrition among vulnerable ethnic groups, because benefits related with breastfeeding are higher than the risk of mercury-exposure. These benefits have been shown to be protective against morbidity and mortality as well as related to the promotion of optimal physical and cognitive development, and the reduction of the risk of contracting some chronic diseases related to nutrition in adulthood ^10^.

Otherwise, selenium is an antagonist of mercury toxicity. According to some researchers, the mechanisms involved in this protection are related to a direct effect on the absorption, disposal, and removal of mercury. Some studies have shown that selenium can prevent mercury poisoning ^11^. Mercury has a strong binding affinity for selenium and works by binding to selenium, producing mercury selenium, a non-absorbable substance in the human body ^12^. As a result of this new compound, the body eliminates mercury before it settles in fatty tissue and causes damage. This interaction between mercury and selenium prevents effective mercury uptake.

However, the interaction between selenium and mercury is complex and depends on several factors, including the chemical forms of selenium and mercury, the relative amounts of each, and the organ in which they interact ^13^. For instance, in a study conducted by Orct et al. with lactating rats exposed simultaneously to selenium and mercury (II) for 4 days, it was found that the concentration of mercury in organs and plasma decreased with the highest oral doses of selenium ^14^. The recommended selenium intake for children and adolescents is 15-40 μg/day and for adults 19-70 years is 55 μg/day. It has been found that a dose of 200 μg/day can neutralize the toxic effects of mercury ^15^.

A review of international data from 28 countries reported that serum/ plasma selenium levels were 91.51 ± 18.78 μg/L among adults ^16^, whereas a study with apparently healthy people living in Turbo or El Bagre (Antioquia) reported that mean plasma selenium was 43.5 ± 12.7 μg/L (95% CI: 39.5 to 47.5) ^17^. These concentrations are much lower than the international mean, which would allow an intervention by increasing concentrations. However, the importance of the selenium as chelating agent ^18^ has not been very studied in Colombia, despite the geological importance of this element.

On the other side of the spectrum, the adverse effects of selenium on the ground and some vegetables were described by some chroniclers during the time of the Conquest (16^th^ and 17^th^ centuries). Fray Pedro Simón described the toxicity of corn and other vegetables that grew in specific places; poisoned animals and humans fell their hair and had dermal lesions ^19^. Subsequent geological studies confirmed the presence of selenium at very high levels in various regions of the Colombian Eastern Cordillera ^20^. Even recent studies in several Colombian mining regions have found that selenium has protective effects on some metals at relatively low doses, and adverse effects when selenium has high concentrations and acts synergistically with other metals ^21^^,^^22^. This means that there is no single recipe to use selenium in the prevention of mercury-related adverse effects among Colombian peoples.

In this context, the objective of this study was to identify potentially useful selenium-rich foods to control the levels of mercury among a group of Afro-Colombians. This approach seeks to have an appropriate intercultural approach that allows long-term effectiveness, respecting the traditions of ethnic groups, and without generating additional costs. For a food to be important in a diet-based intervention, it is important to consider the content of selenium in the food, the frequency of consumption, and the “food culture”. The content is an inherent characteristic of the food, which could be modified by adding selenium, as currently occurs in Colombian products such as bread and eggs, available in various regions of the country. The frequency of consumption is an indicator of the availability, taste of the food, customs, and preferences, among the population, so they correspond to the first option of being included in a diet-based intervention. Then, future specific food intervention strategies could be considered, such as nutritional education focused on the incorporation of foods with a high selenium content or the fortification of foods with this micronutrient.

## Material and methods

**Figure 1:**
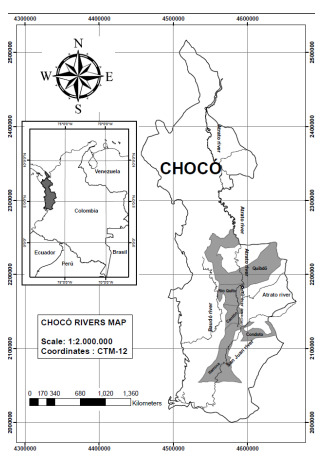
Municipalities included in the study (Chocó, Colombia)

This study was conducted in the municipalities of Quibdó, Río Quito, Cantón, Itsmina, and Condoto, all in the Chocó department (figure 1). This department is in the northwest of the country being border with Panamá. This region is one of the most biodiverse and rainiest in the world (average annual precipitation: 8,000 to 13,000 mm) ^23^, and its geography is dominated by extensive jungles and the basins of the Atrato, San Juan and Baudó rivers.

In Chocó there is gold and platinum mining (a high proportion of this activity is informal and illegal) in a context where there is illegal armed groups and criminal organizations ^24^. In this way it is a clear example of a region where gold mining is associated with poverty and other manifestations of social inequality ^25^. The five municipalities were selected because of the high number of workers involved in artisanal gold mining.

The department of Chocó is the one with the highest concentration of Afro population in Colombia. This population began to arrive in Colombian territory since the times of the Conquest, in the 16th century. Gold was the reason that led to the appearance of settlements, and the mining extraction of the gold present in the rivers was the main reason for the Afro to arrive in the region. However, in this jungle region there were multiple Afro and indigenous revolts that prevented large-scale mining until the middle of the 17th century. The possession of slaves was a sign of power and wealth, which ended after the Independence from Spain (1810) and the entry into force of the abolition of slavery in 1851 ^26^.

Freedom allowed the various Afro communities from Chocó and those who immigrated from other regions to mix and disperse throughout the jungle regions, where they began to have subsistence activities, such as fishing, agriculture, and in some cases artisanal gold mining. The isolation of the Andean regions of the country, from where political and economic power is exercised, allowed the emergence of an Afro culture where colonialism, slavery, Catholic religion, African knowledge and traditions were mixed ^27^. This together with the government abandonment of this region of the country generated a context of very high social vulnerability. Recent econometric studies show that large-scale slavery during the Colonial period is associated with lower gross domestic product and greater poverty today ^28^. Without a doubt, these are some of the social determinants with more influence on health conditions of the Afro-Colombian population.

### Study design, participants, and data collection

A cross-sectional study with individuals over 18 years of age, who had been living in the region for at least 6 months, was carried out. The participants were volunteers selected for convenience. This methodological option was the only possible because in Colombia has no real data on the number of people engaged in informal mining. Moreover, people who carry out these activities frequently move from one place to another, and they are afraid to provide information. All participants were informed about the objectives of the study, which facilitated the collaboration of the community during the field work.

Through questionnaire the researchers inquired about gender (male or female), age (years), place of residence (the five municipalities), education (illiterate, primary, secondary, technical and university), prior poisoning with mercury (yes or not), current occupation with exposure to mercury (yes or not), cigarette consumption (yes or not), and alcoholic beverage consumption (yes or not). To define the foods that would be included in the analysis, the main dishes of the region ^29^, and studies with the Afro-Colombian population of Chocó were reviewed ^30^^,^^31^, and verified in field by two researchers. Additionally, the foods that specialized literature identifies with greater selenium content were reviewed. Thus, frequency of consumption (never, monthly, weekly or diary) of guava, spinach, strawberries, sunflower seeds and walnuts, fish consumption (yes or not) was obtained from each participant. This type of abbreviated food frequency questionnaire was culturally appropriate for the population studied.

### Mercury concentrations in hair

Hair samples were taken from the scalp of occipital region (~10 mg per participant). The samples were stored in polyethylene bags at room temperature. Total mercury was determined according to EPA method 7473 (thermal decomposition, amalgamation and atomic absorption spectroscopy) ^32^ and analyzed in the DMA-80 TriCell Milestone equipment at the Colombian Instituto Nacional de Salud. The environmental health laboratory has a rigorous internal laboratory quality system, and it also participates in an external quality program with the Centre de Toxicologie du Québec, Canada. The limit of quantification was 0.09 μg/g and the limit of detection was 0.026 µg/g.

### Statistical methods

Description of categorical variables was realized with percentages, and continuous variables with central tendency and dispersion measures, according to the distribution observed with the Shapiro-Wilk test. The association between variables was expressed by coefficients obtained in simple and robust regressions, which included the variables of frequency of food consumption as independent, and mercury in hair as a dependent variable. The analysis was realized with Stata 17™ (Stata Corporation, College Station, USA).

### Ethical considerations

This study followed the international ethical principles regarding research with humans, and the Colombian legislation for health research with humans (Resolution 8430 of 1993 of the Ministry of Health). The Ethics Committee of the Instituto Nacional de Salud of Colombia approved the proposal (#36/2015).

## Results

Table 1 shows the characteristics of the participants in the study. It is observed that 85.94% are women and that the median age of the participants was 46 years (between 18 and 98 years old). Of the participants, 41.88% were illiterate, with a marked difference concerning the other educational levels. Many people reported having been previously intoxicated with mercury due to their work activity, which is related to the use of this metal through “barequeo” (activity of washing the sand in a tray or “barequera” to extract gold) for artisanal gold extraction, mainly an activity of women. High mercury concentrations were found in hair, which exceeded up to 39 times the permissible limit in one extreme case.

**Table 1:** Characteristics of participants in the study (n=320)

Variable		n	%
Sex (female)		275	85.94
Age (years)			
	Median	46	
	Q_75_-Q_25_	23	
	min-max	18 - 98	
Place of residence			
	Quibdó	61	19.06
	Río Quito	86	26.88
	Cantón	42	13.13
	Istmina	70	21.88
	Condoto	61	19.06
Education			
	Illiterate	134	41.88
	Primary	61	19.06
	Secondary	80	25.00
	Technical	24	7.50
	University	21	6.56
Occupation with mercury exposure		262	52.19
Previous mercury poisoning self-reported		122	38.13
Cigarette consumption		121	37.81
Alcoholic beverage consumption		168	52.50
Fish consumption		287	89.69
Mercury concentration in hair (µg/g)			
	Median	0.407	
	Q_75_-Q_25_	0.883	
	min-max	0.006 - 40.028	

The frequency of selected selenium-rich food consumption is observed in table 2. Note that the main foods with selenium consumed daily were guava, strawberries, spinach, sunflower seeds, and walnuts. In the opposite, those with less consumption were broccoli, sunflower seeds, and puffed wheat. The association between mercury in hair and consumption of strawberries, sunflower seeds, walnuts, whole wheat flour and yeast extract is in table 3. No associations with other foods were observed. The only gradient protective crude association was observed with the strawberries. In the adjusted models, only protective associations with the monthly consumption of walnuts, whole wheat flour, and yeast extract were observed. This is perhaps a consequence of the low proportion of individuals with high consumption of these foods.

**Table 2:** Frequency of consumption of selenium-rich foods explored in this study (n=320)

Food	Consumption (%)			
	Never	Monthly	Weekly	Diary
Broccoli	82.50	8.13	8.44	0.94
Guava	33.13	32.19	25.31	9.38
Heart (cow)	67.81	16.56	13.13	2.50
Kidney (cow)	67.81	16.25	13.44	2.50
Liver (cow)	42.81	30.00	24.69	2.50
Puffed wheat	81.25	7.19	7.81	3.75
Spinach	61.25	15.00	18.13	5.63
Strawberries	46.88	25.00	20.00	8.13
Sunflower seeds	82.50	5.31	6.88	5.31
Walnuts	78.75	9.06	6.88	5.31
Whole wheat flour	43.13	28.13	26.88	1.88
Yeast extract	48.13	29.38	20.31	2.19

**Table 3:** Association between mercury hair concentration and consumption of selenium-rich foods explored in this study (n=320).

Food	Consumption	β	95% CI	Adj. β*	95% CI
Strawberries	Never	Ref			
	Monthly	-0.02	(-0.16; 0,12)	0.05	(-0.09; 0.19)
	Weekly	-0.17	(-0.32; -0.01)	-0.10	(-0.25; 0.05)
	Daily	-0.24	(-0.46; -0.02)	-0.03	(-0.27; 0.21)
Sunflower seeds	Never	Ref			
	Monthly	-0.23	(-0.49; 0.03)	-0.14	(-0.41; 0.13)
	Weekly	-0.29	(-0.52; -0.06)	-0.16	(-0.45; 0.12)
	Daily	-0.23	(-0.49; 0.03)	-0.89	(-0.41; 0.23)
Walnuts	Never	Ref			
	Monthly	-0.34	(-0.55; -0.14)	-0.22	(-0.43; -0.01)
	Weekly	-0.16	(-0.39; 0.07)	-0.04	(-0.27; 0.20)
	Daily	-0.19	(-0.45; 0.07)	-0.07	(-0.35; 0.22)
Whole wheat flour	Never	Ref			
	Monthly	-0.24	(-0.38; -0.10)	-0.14	(-0.28; -0.01)
	Weekly	-0.14	(-0.28; 0.00)	-0.01	(-0.15; 0.13)
	Daily	-0.24	(-0.67; 0.19)	-0.31	(-0.72; 0.09)
Yeast extract	Never	Ref			
	Monthly	-0.22	(-0.36; -0.09)	-0.15	(-0.28; -0.01)
	Weekly	-0.23	(-0.38; -0.07)	-0.06	(-0.22; 0.09)
	Daily	-0.16	(-0.55; 0,23)	-0.21	(-0.58; 0.17)

* Adjusted per sex, age, fish consumption, locality and education

## Discussion

Findings of this study suggest that walnuts, whole wheat flour and yeast extract can be used to diminish the levels of mercury among Afro-Colombian individuals exposed in mining activities. Although their consumption is not frequent among this population, the fact that they include these foods in their diet is a good starting point for future dietary interventions. Some traditional foods that use nuts are immature banana juice (jugo de guineo), pineapple colada (colada de piña) and borojó (B. patinoi) sorbet (sorbete de borojó); the traditional cookies cucas (made of wheat flour, panela derived from sugar cane, water, eggs, cinnamon baking powder), and the enyucado (cake based on grated cassava) includes nuts and yeast among its ingredients ^29^. With these foods it is possible to begin a diet rich in selenium, increasing its content and improving its flavor. Note that selenium-rich foods that may also have high concentrations of mercury, nor foods from other regions that involve spending additional money have not been included.

However, restrictions in the consumption of mercury-rich foods can be included in the diets when there is no risk of causing nutritional deficiencies. In this regard, the consumption of carnivorous fish that have shown higher concentrations of mercury should be avoided ^33^^-^^35^. The exclusion of breast milk in the diet of neonates and young children ^8^ should not be a generalized option and should only be reserved for cases of severe intoxication, and under supervision of health professionals. Deficiencies in micronutrients such as selenium and zinc can increase susceptibility to the adverse effects of mercury and other toxics. Conversely, a diet rich in antioxidants, such as vitamins C and E, can reduce oxidative stress caused by mercury. For this reason, the importance of adequate nutritional status based on a varied and balanced diet, which contributes to reducing the susceptibility of people to the toxic effects of mercury ^36^.

Food culture is made up of eating habits, what we eat, when and how we eat. Considering the food culture of populations, especially minorities such as indigenous or Afro-descendant population, is necessary to facilitate that food intervention are successful ^37^. For the population health practice, it is essential to understand the food culture in particular settings and the complex elements that are related to it, in such a way that food interventions can contribute to improving the health and nutritional status of populations ^38^.

Those interventions could be based on interculturality and respect for diversity. Factors such as illegal mining, drug trafficking and the presence of armed illegal groups have a negative influence on the nutritional status of the Chocó communities. In Colombia, some studies have been carried out related to eating habits and nutritional status in the Chocó population ^39^^-^^44^, most of them carried out with children. There are no studies on differential food interventions for the adult Afro-descendant population of Chocó.

This study should be understood with some limitations. The main ones are the cross-sectional design used and the possible confusion due to variables not included that prevent defining causality. The sample was not representative of the Afro-Colombian population in Chocó, but we consider it sufficient to explore selenium-rich foods potentially useful in future interventions.

Additionally, in this study we did not ask about other selenium-rich foods such as fish, shellfish, red meat, eggs, chicken, dairy products and derivatives, or grains and cereals because these would not be included in a culturally adapted intervention. Some of these foods accumulate a lot of mercury and others are not traditional food of the region, which would lead to a cost that would not allow the intervention to be maintained in the medium and long term.

Another limitation was the way of measuring food consumption was not detailed enough, and it is possible that it has measurement errors, which are expressed in the lack of an exposure gradient. However, we consider that this approach could be used as a basis to identify the basic elements of an intervention based on diet in this population group. Further intervention studies could include diets based on our findings and include serum/plasma selenium levels among the participants, to find the amount of selenium- rich food required to achieve the chelating action in this population, without increasing selenium to levels that lead to toxic effects. Reducing exposure to mercury in the population, especially in those groups that live in contaminated areas and in people considered susceptible (children, pregnant and lactating women) should represent a priority for health authorities and for decisionmaking in public health.

In conclusion, this study provides culturally acceptable evidence from Afro-Colombian populations for actions that aim to control environmental exposure to mercury through the consumption of selenium-rich food. Further studies will be able to explore in greater depth the number of selenium-rich foods required to control mercury levels in the population participating in this study and use similar methodologies with other vulnerable groups to define the best culturally accepted strategies to reduce mercury concentrations.
